# Draft genomes of *Parafrankia* sp. strains FMc2 and FMc6, isolated from root nodules of *Morella cordifolia*

**DOI:** 10.1128/mra.01270-24

**Published:** 2025-03-25

**Authors:** Dale A. Wilcox, Tyrone Genade

**Affiliations:** 1 Department of Medical Education, J. H. Quillen College of Medicine, East Tennessee State University 4154, Johnson City, Tennessee, USA; The University of Arizona, Tucson, Arizona, USA

**Keywords:** *Frankia*, Parafrankia, *Morella*, Cape flora

## Abstract

The genomes of two *Parafrankia* strains isolated from *Morella cordifolia* root nodules are described. Strain FMc2 is most closely related to *Parafrankia* strain Cc1.17, while FMc6 is a strain of *Parafrankia soli*.

## ANNOUNCEMENT

The family *Frankiaceae* contains free-living soil bacteria which form nitrogen-fixing symbiosis with actinorhizal plant hosts from eight families (1, 2). This symbiosis contributes to nitrogen reservoirs in terrestrial ecosystems and allows actinorhizal plants to perform as pioneers (3, 4). Taxonomic revision has expanded the number of genera within the family from one (*Frankia*) to four, containing strains from phylogenetic clusters 1, 2, 3, and 4 (5), broadly overlapping their associations with different host genera (1, 3, 4, 6). *Morella* represents more than half of the actinorhizal species in Africa (7, 8), forming effective symbioses with strains of both *Frankia* and *Parafrankia* (formerly *Frankia* clusters 1 and 3, respectively) (9). We describe genomes of two *Parafrankia* sp. strains isolated from *Morella cordifolia* (10, 11). Strains FMc2 and FMc6 were sequenced to provide information on *Parafrankia* diversity and selected from 10 strains isolated from African *Morella* (10, 11), based on 16S rRNA, 23S ITS rRNA, and *nifH* gene sequences, multilocus sequence analysis (MLSA), and phenotypic characterization (10–15). For all 10 isolates, partial sequences were deposited in GenBank under accession numbers KP342101-KP342110 (16S rRNA), KP342111-KP342120 (*nifH*), and KU174954-KU174963 (23S ITS rRNA). Gene fragments from MLSA were deposited under accession numbers KU174964-KU174973 (*atpD*), KU174974- KU174983 (*dnaA*), KU174984-KU174993 (*ftsZ*), KU174994-KU175003 (pgk), and KU175004-KU175013 (rpoB).

*Parafrankia* sp. strains FMc2 and FMc6 grew in the dark at 28°C in liquid defined propionate medium for 90 days and gDNA was extracted from single colonies using the CTAB method (16). Standard shotgun libraries were created using a Nextera XT DNA Library Prep kit and 3.0 Illumina chemistry (17). Sequencing was performed at the University of the Western Cape sequencing facility using an Illumina MiSeq platform (2 × 300 bp). For FMc2, sequencing generated 9,137,722 reads totaling 2,435 Mbp. For FMc6, sequencing generated 3,939,506 reads totaling 990 Mbp. Sequence reads were filtered and trimmed using fastp (18). Reads with an average %GC <54 were removed with BBDuk (https://jgi.doe.gov/data-and-tools/bbtools/bb-tools-user-guide/). Genomes were assembled using SPAdes 3.15.5 (19), and quality was assessed with BUSCO v5.7.1 (20) and CheckM v1.2.3 (21). Contigs shorter than 500 bp were discarded. Genome statistics were calculated using BBTools (https://jgi.doe.gov/data-and-tools/bbtools/bb-tools-user-guide/), summary statistics are presented in Table 1. Default parameters were used unless stated otherwise.

**TABLE 1:** *Parafrankia* sp. genome information

Strain	Genome coverage	Number of scaffolds	N50 value (Kb)	Total length (bp)	GC (%)	Completeness^*a*^	Contamination^*b*^
FMc2	32×	533	53.3	8,990,676	71.6	95.8%	1.29%
FMc6	17×	671	28.3	8,928,102	71.3	94.9%	0.83%

^*a*^ Estimated using BUSCO.

^*b*^ Estimated using checkM.

Draft genomes were annotated via the NCBI Prokaryotic Genome Annotation Pipeline (PGAP) (22). *Parafrankia* sp. strain FMc2 contained 7,602 protein-encoding genes, 48 tRNAs, 2 complete rRNA regions, and 2 noncoding RNAs. *Parafrankia* sp. strain FMc6 contained 7,764 protein-encoding genes, 46 tRNAs, 2 complete rRNA regions, and 2 noncoding RNAs. The genome size and number of genes for both strains fall within the expected range for *Parafrankia* (5).

Whole-genome taxonomic analysis was performed via the Type Strain Genome Server (TYGS) platform (23), including digital DNA-DNA hybridization (dDDH) estimation in comparison to other *Parafrankia* genomes. Average nucleotide identity (ANI) was calculated using pyani v0.2.12 (24). Analyses included ten *Parafrankia* strains available from the NCBI genome database. Using a 70% dDDH species threshold, FMc6 was identified as a strain of *Parafrankia soli* (dDDH value 81.9%, ANI value 0.976), whereas FMc2 is most closely related to *Parafrankia colletiae* strain Cc1.17 (dDDH value 55.9%, ANI value 0.935). Bioinformatic analysis using antiSMASH (25) revealed abundant secondary metabolite biosynthetic gene clusters, as seen in other *Parafrankia* species (26, 27 ).

## Data Availability

Whole Genome Shotgun projects were deposited in DDBJ/ENA/GenBank under accession no. JBFLUG000000000 (Parafrankia sp. FMc2) and JBFLUH000000000 (Parafrankia sp. FMc6). Genomes described in this paper are the first versions, JBFLUG01000000.1 and JBFLUH01000000.1. Assemblies and raw reads are available under BioProject number PRJNA1109318. SRA accessions are SRX25638426 (Parafrankia sp. FMc2) and SRX25638427 (Parafrankia sp. FMc6).
